# Clinical Profiles and Mortality of COVID‐19 Inpatients with Parkinson's Disease in Germany

**DOI:** 10.1002/mds.28586

**Published:** 2021-05-04

**Authors:** Raphael Scherbaum, Eun Hae Kwon, Daniel Richter, Dirk Bartig, Ralf Gold, Christos Krogias, Lars Tönges

**Affiliations:** ^1^ Department of Neurology, St. Josef‐Hospital Ruhr‐University Bochum Bochum Germany; ^2^ DRG MARKET Osnabrück Germany; ^3^ Neurodegeneration Research, Protein Research Unit Ruhr (PURE) Ruhr University Bochum Bochum Germany

**Keywords:** Parkinson's disease, epidemiology, hospitalization, COVID‐19 risk factors, COVID‐19 mortality

## Abstract

**Background:**

Comprehensive, nationwide data regarding Parkinson's disease (PD) hospitalizations, coronavirus disease 2019 (COVID‐19) in‐hospital frequency, and COVID‐19‐associated inpatient mortality during the first wave of the COVID‐19 pandemic are not available.

**Objective:**

To provide a nationwide analysis on hospitalized PD patients in Germany and evaluate the impact of the COVID‐19 pandemic.

**Methods:**

We conducted a cross‐sectional study using an administrative claims database covering 1468 hospitals and 5,210,432 patient hospitalizations including a total of 30,872 COVID‐19^+^ cases between January 16 and May 15, 2020.

**Results:**

Compared to 2019, hospitalizations for PD transiently decreased by up to 72.7% in 2020. COVID‐19 frequency was significantly higher in the population of 64,434 PD patients (693 being COVID‐19^+^) than in non‐PD patients (1.1% vs. 0.6%, *P* < 0.001), especially in subjects with advanced age (≥ 65 years). Regarding established COVID‐19 risk comorbidities, COVID‐19^+^ inpatients with PD showed higher incidences than non‐PD COVID‐19^+^ subjects, particularly hypertension and chronic kidney disease. Advanced age and male sex were significantly more frequent in COVID‐19^+^ than in COVID‐19^−^ PD patients. The COVID‐19 inpatient mortality rate was much higher in PD patients than in non‐PD patients (35.4% vs. 20.7%, *P* < 0.001), especially in patients aged 75–79 years. Of note, overall inpatient mortality of PD patients was significantly higher in 2020 than in 2019 (5.7% vs. 4.9%, *P* < 0.001).

**Conclusions:**

PD inpatients are more frequently affected by COVID‐19 and suffer from increased COVID‐19‐associated mortality in comparison to non‐PD patients. More comprehensive studies are needed to assess the significance of associated comorbidities for COVID‐19 risk and mortality in PD. © 2021 The Authors. *Movement Disorders* published by Wiley Periodicals LLC on behalf of International Parkinson and Movement Disorder Society

## 1 Introduction

Since December 2019, our global society has been influenced by coronavirus disease 2019 (COVID‐19) which is caused by the novel severe acute respiratory syndrome coronavirus 2 (SARS‐CoV‐2). As of February 18, 2021, approximately 110 million confirmed COVID‐19 cases and 2.4 million deaths have been reported globally.^1^ Apart from frequent respiratory symptoms in COVID‐19 patients, neurologic manifestations occur in about 80% of hospitalized cases at any stage of the disease course.^2^ Neurologic complications such as encephalitis, stroke, muscular damage, or anosmia^3^ are associated with increased mortality risk.^4^ Besides, it is currently under discussion whether pre‐existent neurologic disorders can deteriorate under COVID‐19.

The first wave of the COVID‐19 pandemic in the first half of 2020 has indirectly and directly affected patients with Parkinson's disease (PD). Indirect effects comprise a large impact on health care provision including a reduction in overall hospitalizations. As of March 2020, Germany was one of the most severely affected countries worldwide, and because of the need for more COVID‐19 treatment capacities, (semi‐)elective hospital treatments were postponed or even cancelled. However, chronic illnesses require an extensive service of care including hospitalizations in cases of exacerbation of primary neurologic disease or because of worsened comorbidities. The numbers of patients who had to be hospitalized for treatment of PD or concomitant disease has strongly risen in the last year as we have shown in our own recent analysis of the entire German population.^5^ Direct effects of COVID‐19 on PD were those that led to significant worsening of motor and nonmotor symptoms which were attributed to both infection‐related mechanisms and impaired pharmacokinetics of dopaminergic therapy.^6^ Various publications have shown that patients suffering from PD are significantly affected by the COVID‐19 pandemic and perceive it with concern.^7^, ^8^

Risk factors for a severe course of COVID‐19 and mortality are known for the general population. They include general features such as older age^9^, ^10^ or male sex^10^ as well as comorbidities such as hypertension, diabetes, coronary heart disease^9^, ^11^ and respiratory diseases.^10^ For PD populations, several studies indicate that COVID‐19‐positive (COVID‐19^+^) PD patients similarly more frequently present comorbidities such as hypertension,^12^ diabetes,^12^, ^13^ chronic obstructive pulmonary disease (COPD),^14^ obesity,^14^ as well as dementia^13^ if compared to COVID‐19‐negative (COVID‐19^−^) PD patients. Use of advanced therapies, long disease duration, and older age were common,^15^ although there exist conflicting data with respect to age^6^, ^14^ and disease duration.^6^ However, in these studies, sample sizes were rather small, control groups were mostly not included, and disease stage distributions were heterogeneous.

Mortality rates in COVID‐19^+^ PD patients range from 5.7%^14^ to 40%.^15^ COVID‐19^+^ non‐survivors with PD had longer disease duration, concomitant dementia, and often arterial hypertension.^9^ With respect to the high prevalence of age‐related comorbidities in PD patients,^16^, ^17^ it was concluded that a putatively higher COVID‐19 mortality risk in PD is associated with late‐stage PD patients' frailty rather than with PD itself.^14^

In Germany, the first COVID‐19 case was reported on January 28, 2020^1^, ^18^ and the first two related deaths occurred on March 9.^1^, ^18^ Two days later, the World Health Organization (WHO) declared COVID‐19 a pandemic^19^ and Germany was one of the most severely affected countries worldwide at that time. A lockdown comprising social distancing measures and reduction of economic and public life started on March 22^20^ and was loosened from May 6, 2020 onwards.^21^ As a result, the number of new infections decreased from mid‐April onwards.^1^, ^18^

In order to thoroughly assess the situation of hospitalized PD patients during the COVID‐19 first pandemic wave, evaluate disease profiles in comparison to non‐PD and COVID‐19^−^ PD patients, and provide data on the inpatient mortality of COVID‐19^+^ and COVID‐19^−^ PD patients, we performed a nationwide study using an administrative database for the 4‐month period between January 16 and May 15, 2020. We focused our analysis on three principal questions: How did hospitalizations of PD develop during the pandemic? Are PD patients particularly affected by COVID‐19 and what are the characteristics of PD patients diagnosed with COVID‐19? Are PD patients more at risk of COVID‐19‐associated death?

## 2 Methods

### 2.1 Study Design

To describe hospitalization numbers of patients with PD during the first wave of the COVID‐19 pandemic in Germany and to calculate COVID‐19 frequency and inpatient mortality in PD, we conducted a cross‐sectional observational study based on comprehensive nationwide administrative data in Germany between January 16 and May 15, 2020 and 2019, respectively.

### 2.2 Database

We analyzed case numbers of hospital admissions during the first wave of the COVID‐19 pandemic in 2020 on a nationwide level using a high‐quality and validated administrative diagnosis‐related group (DRG) database (data retrieval according to §21 KHEntgG and §24 Abs. 2 KHG; official data on file, source: Institut für das Entgeltsystem im Krankenhaus, InEK, www.g-drg.de). In Germany, all inpatient cases are encoded according to the International Classification of Diseases 10th revision, German Modification (ICD‐10‐GM). Within the DRG coding system, main diagnoses (reason for hospitalization) and secondary diagnoses (comorbidities) are shown. The database covers 1468 hospitals and almost 100% of inpatient cases. As of March 31, 2020, the number of inhabitants of the covered area was 83,157,201.^22^ About 20% of cases are assessed for validity by board‐certified physicians of the medical service of Germany's National Association of Statutory Health Insurance Funds, thus warranting the high quality of the data. Data were retrieved retrospectively on October 17 and 19, 2020 and January 31, 2021 as well as on February 17, 2021.

### 2.3 Outcomes

The primary outcomes were COVID‐19 in‐hospital frequency and inpatient mortality. Secondary outcomes included the relative change in case numbers in 2020 versus 2019, as well as information on age and frequencies of sex, comorbidities, and PD disease severity stages as measured by the Hoehn and Yahr (HY) scale. The unit of analysis for frequency analyses is “case”. Multiple counting was avoided using key “06” (discharge to another hospital). We consider case numbers as patient numbers, since the number of potentially readmitted patients in the examined period is regarded as negligible. As the PD inpatient population is selective due to hospitalization, we report COVID‐19 frequency rather than prevalence, a term used for more defined populations.

We calculated the change in case numbers in the first 4 months of 2020 versus 2019 (between January 16 and May 15, 2020 and 2019, respectively), thereby including the date of the first reported COVID‐19 case in Germany, and the beginning and end of a lockdown period. Additionally, we determined relative differences in case numbers between the first and the second half of March and April 2020, respectively. Here, we determined the number of hospitalizations using the main diagnosis codes for PD and additionally for all further ICD diagnoses, and using the secondary diagnosis codes for laboratory‐confirmed COVID‐19 (U07.1) (Table S1). The database does not provide information on the point of time of COVID‐19 diagnosis but is based on reverse transcription‐polymerase chain reaction (RT‐PCR) proof from a nasopharyngeal swab. For year‐wise comparison of hospitalizations, one additional day was included in 2020 (February 29).

For the analysis of COVID‐19 frequencies and inpatient mortality, we assessed main and secondary diagnosis codes regardless of the reason for hospitalization. Within the inpatient population, we compared the COVID‐19 frequencies of PD and non‐PD patients in distinct age groups and calculated odds ratios (ORs) for COVID‐19 diagnosis. To improve readability, patients with or without laboratory‐confirmed COVID‐19 are termed “COVID‐19^+^” and “COVID‐19^−^”, respectively.

Inpatient mortality analyses were performed applying discharge key “07” (death during hospital stay). Cases with key “07” were termed “non‐survivors”, whereas cases without key “07” were termed “survivors”. We calculated the COVID‐19‐associated inpatient mortality rate in PD and non‐PD patients as the proportion of deaths among the respective population.

### 2.4 Statistical Analysis

For descriptive analyses of the study population, values were calculated as mean and standard deviation (SD) for continuous variables and as absolute (n) and relative (%) frequencies for categorical variables. Differences were assessed by unpaired t‐test and chi‐squared test for continuous and categorical variables, respectively. In order to describe COVID‐19 diagnosis odds and inpatient mortality risk, ORs with 95% confidence intervals (95% CIs) were determined. Due to the short‐term supply of the COVID‐19 data by the German administration and due to database structure, which gives frequencies only and no individual data, adjustment was calculable for age only. Individual data to perform a multivariate analysis accounting for comorbidities as possible confounders were not available. Age as a confounder variable was controlled for by using age group stratification. Significance level was set at 0.05. Data were analyzed using Microsoft Excel for Windows.

## 3 Results

### 3.1 Development of Hospitalization Numbers for PD During the COVID‐19 Pandemic

**FIG. 1:**
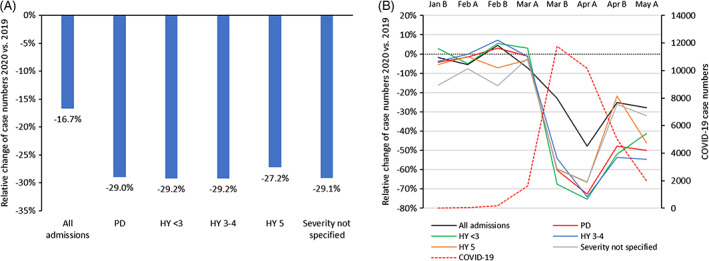
Relative change of case numbers of Parkinson's disease including disease severity, and COVID‐19 case numbers between January 16 and May 15, 2020. (**A**) Overall time period comparison 2020 versus 2019. (**B**) Semi‐monthly comparison 2020 versus 2019. Jan B = January 16–January 31, 2020, Feb A = February 01–February 15, 2020. PD, Parkinson's disease; HY, Hoehn and Yahr; COVID‐19, coronavirus disease 2019. [Color figure can be viewed at wileyonlinelibrary.com]

Overall numbers of hospitalized patients substantially decreased from 6,254,091 to 5,210,432 (−16.7%) in 2020 as compared to 2019 in the period under study between January 16 and May 15 (Fig. 1A and Table S2). Patients with PD as the primary reason for hospitalization were even less frequently admitted in 2020 (15,854 in 2019, 11,262 in 2020, −29.0%). PD cases with HY stages HY<3 and HY 3‐4 decreased slightly more (each −29.2%) than the PD average, while those with HY 5 decreased least (−27.2%).

In the more detailed analysis of semi‐monthly hospitalization numbers, these changed differentially between mid‐January and mid‐May 2020 for PD and for COVID‐19 if compared to 2019 (Fig. 1B and Table S3). The most drastic decrease in PD hospitalization numbers was observed in the first half of April 2020 (−72.7%). COVID‐19 case numbers rose most prominently between the first and the second half of March 2020. If compared to the first half of March 2020, primarily PD‐related hospitalizations decreased substantially (−60.3%) in the second half of March 2020 and showed a much greater reduction than overall hospitalizations (−27.3%). The PD inpatient numbers diminished most for HY stages <3 (−68.4%) and HY 5 (−64.4%), while patients with HY 3‐4 and those with unspecified severity were admitted relatively more continuously and had less significant case reductions (−58.7% and −55.8%, respectively). In line with reduced COVID‐19 hospitalizations between the first and the second half of April, overall PD admissions rose again but did not reach their previous levels (Table S3).

### 3.2 COVID‐19 Frequency in PD Inpatients

Between January 16 and May 15, 2020, a total of 30,872 COVID‐19 inpatient cases were documented in 1468 hospitals in Germany, of which 693 also had PD. COVID‐19 was significantly more frequent in patients with PD than in non‐PD inpatients (1.1% vs. 0.6%, *P* < 0.001). The total inpatient frequency of PD was 1.2%, whereas PD patients represented a total of 2.2% of all COVID‐19 cases.

The higher COVID‐19 frequency in subjects with PD compared to non‐PD patients was particularly prominent in the groups aged 65 years and higher (Table 1). Consistently, bivariate OR calculations revealed higher odds of COVID‐19 diagnosis in these age groups. COVID‐19 frequency in PD patients rises with higher age (Table 1) and more advanced disease (HY <3 0.8%, HY 3‐4 1.0%, HY 5 1.4%; Table S4).

**TABLE 1:** COVID‐19 frequency in age groups of Parkinson's disease (PD) and non‐PD inpatients between January 16 and May 15, 2020. PD, Parkinson's disease; COVID‐19, coronavirus disease 2019; CI, confidence interval; OR, odds ratio; LB, lower bound; UB, upper bound

	Case numbers		COVID‐19 frequency in PD (%)	Case numbers		COVID‐19 frequency in non‐PD (%)	Frequency difference PD vs. non‐PD		COVID‐19 diagnosis odds PD vs. non‐PD		
	PD			Non‐PD					OR	95% CI	
Age group (years)	COVID‐19^+^	COVID‐19^+ and ‐^		COVID‐19^+^	COVID‐19^+ and ‐^		*P*	χ^2^(1)		LB	UB
Total	693	64,434	1.1	30,179	5,145,998	0.6	<0.001	264.341	1.843	1.709	1.988
<30	0	10	0.0	1266	926,197	0.1	0.907	0.014	0.000	0.000	0.000
30–39	0	53	0.0	1411	434,053	0.3	0.678	0.173	0.000	0.000	0.000
40–49	1	317	0.3	2219	359,937	0.6	0.494	0.469	0.510	0.072	3.635
50–54	5	681	0.7	2012	301,185	0.7	0.832	0.045	1.100	0.456	2.654
55–59	7	1417	0.5	2557	386,205	0.7	0.435	0.609	0.745	0.354	1.567
60–64	20	2522	0.8	2573	410,073	0.6	0.292	1.109	1.266	0.814	1.969
65–74	97	12,029	0.8	5180	835,349	0.6	0.009	6.774	1.303	1.065	1.594
75–79	151	14,758	1.0	3811	511,675	0.7	<0.001	15.469	1.378	1.170	1.622
≥80	412	32,647	1.3	9150	981,324	0.9	<0.001	38.388	1.358	1.230	1.500

In order to identify typical characteristics of COVID‐19^+^ PD patients, we compared them to COVID‐19^−^ PD patients as a historical control group from 2019 (Table 2). In terms of COVID‐19 risk factors, COVID‐19^+^ PD patients showed slightly but significantly higher rates of hypertension (54.1% vs. 53.3%, *P* < 0.001) and chronic kidney disease (22.4% vs. 21.0%, *P* < 0.001) than COVID‐19^−^ PD patients. COVID‐19^+^ PD patients were slightly less frequently affected by vitamin D deficiency, cardio‐ and cerebrovascular disease, diabetes type 2, and COPD (Table 2). They more often suffered from advanced PD with HY stage 5, whereas HY stages <5 were significantly less frequent (Table 2). In terms of general characteristics, COVID‐19^+^ PD patients were older (more often aged ≥80 years) and more frequently male than PD inpatients from the same time period in 2019.

**TABLE 2:** Demographic characteristics and comorbidities of all COVID‐19 cases with and without Parkinson's disease (PD), plus PD severity for PD inpatients with and without COVID‐19.

Characteristics and comorbidities	COVID‐19^+^ non‐PD		COVID‐19^+^ PD		COVID‐19^+^ PD survivors		COVID‐19^+^ PD non‐survivors		COVID‐19^−^ PD 2019		*P* value		
											PD vs. non‐PD	Non‐survivors vs. survivors	COVID‐19^+^ vs. COVID‐19^−^ PD
N	30,179		693		448		245		80,794				
Male (n, %)	16,373	54.3%	419	60.5%	258	57.6%	161	65.7%	48,080	59.5%	**0.001**	**0.010**	**<0.001**
Age (M, SD)	67.4	6.9	80.8	13.5	79.4	12.1	82.2	14.9	78.5	11.2	**<0.001**	**0.006**	**<0.001**
<30 years	1266	4.2%	0	0.0%	0	0.0%	0	0.0%	18	0.0%	**<0.001**	n.a.	n.a.
30–39 years	1411	4.7%	0	0.0%	0	0.0%	0	0.0%	50	0.1%	**<0.001**	n.a.	n.a.
40–49 years	2219	7.4%	1	0.1%	1	0.2%	0	0.0%	419	0.5%	**<0.001**	0.459	**<0.001**
50–54 years	2012	6.7%	5	0.7%	5	1.1%	0	0.0%	878	1.1%	**<0.001**	0.096	**<0.001**
55–59 years	2557	8.5%	7	1.0%	6	1.3%	1	0.4%	1699	2.1%	**<0.001**	0.205	**<0.001**
60–64 years	2573	8.5%	20	2.9%	19	4.2%	1	0.4%	3095	3.8%	**<0.001**	**0.003**	**<0.001**
65–74 years	5180	17.2%	97	14.0%	78	17.4%	19	7.8%	15,419	19.1%	**0.027**	**<0.001**	**<0.001**
75–79 years	3811	12.6%	151	21.8%	95	21.2%	56	22.9%	20,121	24.9%	**<0.001**	0.527	**<0.001**
≥80 years	9150	30.3%	412	59.5%	244	54.5%	168	68.6%	39,095	48.4%	**<0.001**	**<0.001**	**<0.001**
Vitamin D deficiency (E55)	444	1.5%	20	2.9%	14	3.1%	6	2.4%	2682	3.3%	**0.002**	0.543	**<0.001**
Hypertension (I10)	13,711	45.4%	375	54.1%	237	52.9%	138	56.3%	43,055	53.3%	**<0.001**	0.283	**<0.001**
Cardiovascular disease (I25)	5082	16.8%	148	21.4%	92	20.5%	56	22.9%	17,636	21.8%	**0.001**	0.368	**0.001**
Cerebrovascular disease (I69)	964	3.2%	32	4.6%	18	4.0%	14	5.7%	3886	4.8%	**0.033**	0.176	**0.009**
Diabetes type 2 (E11)	6607	21.9%	160	23.1%	97	21.7%	63	25.7%	19,084	23.6%	0.447	0.123	**<0.001**
COPD (J44)	1999	6.6%	34	4.9%	19	4.2%	15	6.1%	4857	6.0%	0.069	0.144	**<0.001**
Chronic kidney disease (N18)	5035	16.7%	155	22.4%	87	19.4%	68	27.8%	16,960	21.0%	**<0.001**	**0.001**	**<0.001**
Inpatient mortality	6241	20.7%	245	35.4%	0	0.0%	245	100.0%	4104	5.1%	**<0.001**	n.a.	**<0.001**
HY <3	n.a.	n.a.	80	11.5%	58	12.9%	22	9.0%	12,369	15.3%	n.a.	0.064	**<0.001**
HY 3‐4	n.a.	n.a.	202	29.1%	131	29.2%	71	29.0%	26,878	33.3%	n.a.	0.928	**<0.001**
HY 5	n.a.	n.a.	50	7.2%	27	6.0%	23	9.4%	4406	5.5%	n.a.	**0.027**	**<0.001**
Severity not specified	n.a.	n.a.	361	52.1%	232	51.8%	129	52.7%	37,141	46.0%	n.a.	0.786	**<0.001**

PD, Parkinson's disease; COVID‐19, coronavirus disease 2019; M, mean; SD, standard deviation; COPD, chronic obstructive pulmonary disease; HY, Hoehn and Yahr; n.a., not available. *P* values <0.05 are printed in bold

We further compared the characteristics of COVID‐19^+^ PD and COVID‐19^+^ non‐PD patients (Table 2). COVID‐19^+^ PD patients were significantly older (more often aged ≥75 years), more frequently male, and showed even higher frequencies of COVID‐19 risk factors than COVID‐19^+^ non‐PD patients. More specifically, they more frequently suffered from hypertension, cardio‐ and cerebrovascular disease, chronic kidney disease, and vitamin D deficiency, whereas type 2 diabetes and COPD were similarly frequent in COVID‐19^+^ PD and non‐PD patients (Table 2).

### 3.3 COVID‐19‐Associated Inpatient Mortality in PD

**FIG. 2:**
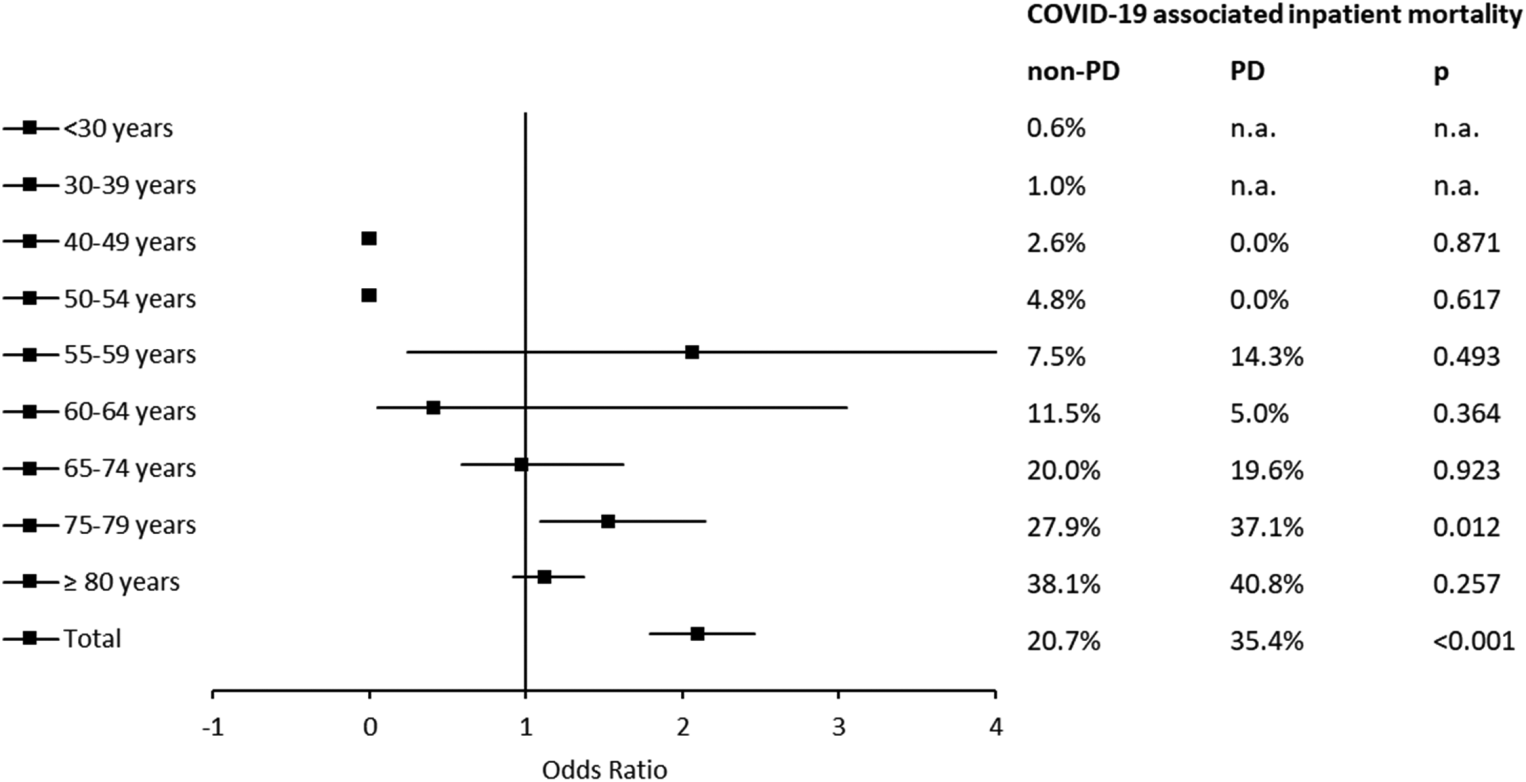
COVID‐19‐associated inpatient mortality in age groups of Parkinson's disease (PD) and non‐PD inpatients between January 16 and May 15, 2020. COVID‐19, coronavirus disease 2019; PD, Parkinson's disease; n.a., not available.

In order to evaluate COVID‐19‐associated inpatient mortality, we calculated the COVID‐19 inpatient mortality rate and compared it between PD and non‐PD patients (Fig. 2, Table S5). Overall COVID‐19‐associated mortality was significantly higher in PD patients (35.4%) compared to all non‐PD hospitalized COVID‐19^+^ patients in Germany (20.7%, *P* < 0.001). For patients aged 75–79 years, the odds to die were approximately 1.5‐fold higher in PD patients (OR 1.526, 95% CI 1.089–2.139, *P* = 0.012). Correspondingly, this age group showed the greatest mortality difference between PD and non‐PD patients. Due to the database structure, adjustment for comorbidities was not calculable.

In order to determine the characteristics of COVID‐19^+^ PD non‐survivors, we compared their demographic and PD features as well as comorbidity rates with COVID‐19^+^ PD survivors (Table 2). With respect to age and gender, patients with fatal outcome were older and significantly more likely to be male. Non‐survivors showed more frequently HY stage 5. In terms of comorbidities, PD inpatients who died with COVID‐19 suffered more frequently from chronic kidney disease.

Furthermore, we compared overall PD inpatient mortality between 2020 and 2019, independent of COVID‐19 status. Overall mortality of PD inpatients was significantly higher in 2020 (5.7% vs. 4.9%, relative difference 16.5%, *P* < 0.001; Table S5), especially in age groups with high COVID‐19 frequencies (≥ 65 years). In particular, patients aged 65–74 years and 75–79 years died more frequently in 2020 (3.3% vs. 2.7%, relative difference 21.9%, *P* < 0.001 and 5.2% vs. 4.3%, relative difference 20.0%, *P* < 0.001, respectively).

## 4 Discussion

This is the first study to analyze administrative data of hospitalized PD patients in Germany during the COVID‐19 pandemic in a comprehensive nationwide approach. We analyzed the dynamics of hospitalization of PD patients during the first wave of the pandemic in 2020, COVID‐19 in‐hospital frequency, and inpatient mortality as well as characteristics of COVID‐19^+^ PD patients.

In relation to the overall decrease of hospitalization numbers in 2020, the decrease of admissions was found to be significantly more pronounced for PD. The most drastic reduction of PD admissions occurred during the first peak of COVID‐19 outbreak between the first and second half of March 2020, particularly for patients with HY stages <3 or 5. From mid‐April, overall COVID‐19 cases decreased and PD inpatient numbers recovered, however, without reaching pre‐pandemic levels.

The social restrictions and preventive measures during the COVID‐19 pandemic have radically impacted the perceptions of patients and healthcare providers on various aspects of daily routine. In a recent online survey, PD patients expressed their concern about contracting COVID‐19 because of their diagnosis, resulting in canceling or postponing medical appointments.^23^ Although the risk of hospital‐acquired COVID‐19 is low,^24^ the threshold for hospital admission was raised in fear of contracting COVID‐19 at hospitals.^7^, ^25^, ^26^, ^27^ Regarding healthcare providers, hospitals were compelled to ensure sufficient intensive care unit (ICU) capacities and postpone elective procedures (including deep brain stimulation or initiation of infusion therapies) if not mandatory and received corresponding subsidies for beds that were kept free.^28^ For less impaired PD patients represented by HY <3, avoidance of hospital care might have been compensated. PD patients in advanced to late disease stage were often treated in isolation at home or in a care facility, thereby cocooning a frail at‐risk population.^29^

In our study, COVID‐19 frequency was significantly higher in hospitalized patients with PD than in the non‐PD inpatient population, especially for patients aged ≥65 years. Concerning overall COVID‐19 prevalence in PD patients, a multicenter study from Tuscany also suggested a higher rate of COVID‐19 in the PD population.^12^ Another study in non‐advanced, community‐dwelling PD patients from Lombardy, Italy,^14^ however, found no difference between PD patients (7.1%) and non‐PD family members (7.6%) in COVID‐19 prevalence.

Within hospitalized COVID‐19 patients, those with PD exhibited more COVID‐19 risk factors (old age, male sex, specific comorbidities) than non‐PD patients. More specifically, they more frequently suffered from hypertension, cardio‐ and cerebrovascular disease, and chronic kidney disease, whereas type 2 diabetes and COPD were similarly frequent in COVID‐19^+^ PD and non‐PD patients. We would like to point out in this respect that higher COVID‐19 frequencies in PD patients might be related to age, sex, and comorbidities rather than to PD per se. A large population study in 2017 demonstrated that PD patients have higher levels of comorbidity including coronary heart disease, cerebrovascular disease, and heart failure.^16^ In our own analysis of hospitalized PD patients from 2018 we described significant frequencies of COVID‐19 risk comorbidities.^17^

Smaller studies with double‐digit case numbers did not detect differences between COVID‐19^+^ and COVID‐19^−^ PD patients in terms of age or severity of disease,^6^, ^30^ while we found an increased COVID‐19 frequency in older and advanced PD patients. This could be due to the higher case numbers examined in our study including 693 COVID‐19^+^ and 63,741 COVID‐19^−^ PD patients.

Overall COVID‐19‐associated inpatient mortality was significantly higher in PD patients than in non‐PD patients in our study. COVID‐19^+^ PD non‐survivors were of older age, suffered more frequently from chronic kidney disease, and exhibited more frequently advanced disease stage (HY 5) when compared to PD survivors. These negative outcome parameters are in line with known risk factors for COVID‐19 mortality.^31^, ^32^ Data on mortality rates vary between studies depending on methodology and sample size. In a recent analysis of the Italian ParkLink cohort, a 30‐day inpatient fatality rate of 35.1% was found not to differ among patients with PD, parkinsonism, and the control population.^33^ A community‐based case–control survey suggested a lower mortality rate (5.7%) that did not differ significantly from the rate in the non‐PD population.^14^ Underestimation by exclusion of patients living in nursing homes or care facilities was acknowledged, although high mortality was documented particularly in residential home patients.^34^ A multicenter case series with 117 COVID‐19^+^ PD patients showed an increased mortality rate of 19.7% in PD patients with COVID‐19, and identified dementia, hypertension, and PD duration as risk factors for severe outcome.^35^ A high mortality rate (40%) was reported in a case series of 10 COVID‐19^+^ PD patients of older age and with longer disease duration.^15^ Here, the mortality rate was even higher (50%) among patients on advanced therapies. Higher mortality rates of COVID‐19^+^ PD patients (OR 1.30, 95% CI 1.13–1.49) independent of age, sex, and race were demonstrated in a large US

epidemiological study.^36^ Thus, the current analyses demonstrate an increased mortality risk for COVID‐19^+^ PD patients, especially if based on large administrative claims data. To what extent distinct comorbidity patterns in PD patients or PD per se could be responsible for this finding remains an open question that can only be answered when individualized patient data are made available.

There is currently no definitive answer as to whether PD per se predisposes for SARS‐CoV‐2 infection. It has been suggested that amantadine and alpha‐synuclein may be protective against COVID‐19,^37^, ^38^ whereas some authors hypothesized that SARS‐CoV‐2 infection can also promote secondary neurodegeneration.^39^ PD patients may be particularly exposed to a higher risk for a severe outcome of COVID‐19 considering the general frailty increasing with age and advanced disease stage. Motor and nonmotor symptoms significantly worsen with COVID‐19 and often require therapy adjustments.^6^ Respiratory dysfunction in PD patients can result from cardiopulmonary comorbidities as well as PD‐associated respiratory muscle weakness, axial rigor manifestation, and dysphagia predisposing for pneumonia, which is the foremost reason for hospital admission and the most common cause of death.^40^

### 4.1 Limitations and Strengths

Certain limitations of this cross‐sectional study need to be acknowledged. It focused on hospitalized patients, which may present with more significant overall disease burden. Thus, there is a risk of selection bias that limits the generalizability of results for the general PD population in Germany. However, the study warrants an accurate analysis of a defined population at risk for comparison. With regard to the quality of documentation of comorbidities, the resulting administrative claims are very carefully controlled for validity by board‐certified physicians of the medical service of Germany's National Association of Statutory Health Insurance Funds in order to ensure a high level of quality.

Large‐scale datasets of COVID‐19 infection rates and clinical profiles of the general PD population are not yet available as a comparison. The currently available data for stationary care treatment do not comprise detailed individualized information and therefore do not permit full appreciation of confounding factors such as comorbid diseases. Thus, higher ORs of COVID‐19 diagnosis or associated death in PD could possibly be related to comorbidities or higher frailty. No analytic inference can be drawn of an independent risk of PD patients for COVID‐19 with the existing data.

To our knowledge, this is the first nationwide analysis of PD inpatient care during the COVID‐19 pandemic. By means of the DRG coding system, full coverage of all hospitalized patients with laboratory‐confirmed COVID‐19 is warranted and has permitted us to perform a very thorough analysis of the PD inpatient population throughout Germany.

### 4.2 Conclusions

The COVID‐19 pandemic strongly affects healthcare provision for PD patients. We here demonstrate that during the first wave of the pandemic in 2020, PD hospitalization numbers decreased substantially. COVID‐19 frequency and COVID‐19‐associated inpatient mortality were higher in older PD patients than in non‐PD subjects of the same age. Therefore, PD inpatients are particularly affected by COVID‐19, even if the higher risk of morbidity or inpatient mortality is probably mediated by higher prevalences of certain COVID‐19 risk comorbidities. It is of special importance that overall inpatient mortality of PD patients was increased too, independent of COVID‐19 status. Care must be taken to ensure that optimal treatment for hospitalized PD patients is always guaranteed, and that potentially competing priorities do not have a negative impact on PD care in these pandemic times.

## Author Roles

(1) Research Project: A. Conception, B. Organization, C. Execution; (2) Statistical Analysis: A. Design, B. Execution, C. Review and Critique; (3) Manuscript: A. Writing the First Draft, B. Review and Critique.

R.S.: 1B, 1C, 2A, 2B, 3A, 3B

E.H.K.: 2B, 3A, 3B

D.R.: 2C, 3B

D.B.: 1A, 1B, 1C, 2C, 3B

R.G.: 3B

C.K.: 2C, 3B

L.T.:1A, 1B, 1C, 2C, 3A, 3B

## Financial Disclosures of All Authors for the Preceding 12 Months

The authors have nothing to disclose. Raphael Scherbaum reports no disclosures. Eun Hae Kwon reports no disclosures. Daniel Richter receives FoRUM grant (K136‐20) of the Ruhr‐University of Bochum. Dirk Bartig has received orders for analysis of the German Diagnosis‐Related Groups system from Boehringer Ingelheim and Sanofi Aventis. Ralf Gold received consultation fees and speaker's honoraria from Bayer Schering, Biogen Idec, MerckSerono, Novartis, Sanofi‐Aventis, and TEVA. He also acknowledges grant support from Bayer Schering, Biogen Idec, MerckSerono, Sanofi‐Aventis, and TEVA. Christos Krogias has received speaker honoraria or travel grants for scientific meetings from Bayer Vital, and Daichii Sankyo. Lars Tönges has received travel funding and/or speaker honoraria from AbbVie, Bayer, Bial, Desitin, GE, UCB, and Zambon and consulted for AbbVie, Bayer, Bial, Desitin, UCB, and Zambon.

## Supporting information


**Table S1** Diagnoses used in the study with corresponding codes according to the International Classification of Diseases 10th revision, German modification (ICD‐10‐GM).
**Table S2**: Case numbers of Parkinson's disease (PD) inpatients (admissions) including disease severity between January 16 and May 15, 2020 vs. 2019.
**Table S3**: Semi‐monthly case numbers of Parkinson's disease (PD) and COVID‐19 between January 16 and May 15, 2020 vs. 2019 and relative difference between the first and the second half of March 2020 and April 2020, respectively.
**Table S4**: COVID‐19 frequencies in Parkinson's disease (PD) inpatients with different disease severity stages between January 16 and May 15, 2020
**Table S5**: COVID‐19‐associated inpatient mortality in age groups of Parkinson's disease (PD) and non‐PD inpatients between January 16 and May 15, 2020.
**Table S6**: Inpatient mortality in Parkinson's disease (PD) inpatients between January 16 and May 15, 2020 vs. 2019
