# Supplementary material for: Clinical Profiles and Mortality of COVID‐19 Inpatients with Parkinson's Disease in Germany
Source: Mov Disord. 2021 May 4;36(5):1049–57. doi: 10.1002/mds.28586 (PMC8207013; doi:10.1002/mds.28586)
Supplement: Supplementary file 1 — Table S1 Diagnoses used in the study with corresponding codes according to the International Classification of Diseases 10th revision, German modification (ICD‐10‐GM). Table S2: Case numbers of Parkinson's disease (PD) inpatients (admissions) including disease severity between January 16 and May 15, 2020 vs. 2019. Table S3: Semi‐monthly case numbers of Parkinson's disease (PD) and COVID‐19 between January 16 and May 15, 2020 vs. 2019 and relative difference between the first and the second half of March 2020 and April 2020, respectively. Table S4: COVID‐19 frequencies in Parkinson's disease (PD) inpatients with different disease severity stages between January 16 and May 15, 2020 Table S5: COVID‐19‐associated inpatient mortality in age groups of Parkinson's disease (PD) and non‐PD inpatients between January 16 and May 15, 2020. Table S6: Inpatient mortality in Parkinson's disease (PD) inpatients between January 16 and May 15, 2020 vs. 2019 [file MDS-36-1049-s001.docx]

**Supplementary material**

„Clinical profiles and mortality of COVID-19 inpatients with Parkinson’s disease in Germany”

by Raphael Scherbaum, Eun Hae Kwon, Daniel Richter, Dirk Bartig, Ralf Gold, Christos Krogias and Lars Tönges submitted to *Movement Disorders*

**Table S1:** Diagnoses with corresponding codes according to the International Classification of Diseases, 10^th^ revision, German modification (ICD-10-GM)

| Code | Diagnosis |
| --- | --- |
| U07.1 | COVID-19, virus identified (confirmed by laboratory testing, RT-PCR) |
| G20 | Parkinson's disease (PD) |
| G20.0- | Parkinson’s disease with no or slight impairment, Hoehn and Yahr stages <3 |
| G20.1- | Parkinson’s disease with moderate to serious impairment, Hoehn and Yahr stages 3-4 |
| G20.2- | Parkinson’s disease with the most serious impairment, Hoehn and Yahr stage 5 |
| G20.9- | Parkinson’s disease with not further defined impairment |

**Table S2:** Case numbers of PD inpatients (admissions) including disease severity between January 16 and May 15, 2020 vs. 2019

| PD ICD-Code | Disease severity | n | |  | Difference | |
| --- | --- | --- | --- | --- | --- | --- |
|  |  | 2019 | 2020 |  | n | % |
| G20 | Any | 15,854 | 11,262 |  | -4,592 | -29.0% |
| G20.0- | HY <3 | 1,973 | 1,397 |  | -576 | -29.2% |
| G20.1- | HY 3-4 | 10,630 | 7,527 |  | -3,103 | -29.2% |
| G20.2- | HY 5 | 1,810 | 1,317 |  | -493 | -27.2% |
| G20.9- | not specified | 1,441 | 1,021 |  | -420 | -29.1% |

**Table S3:** Semi-monthly case numbers of PD and COVID-19 between January 16 and May 15, 2020 vs. 2019 and relative difference between the first and the second half of March 2020 and April 2020, respectively. Jan B = January 16 - January 31, 2020, Feb A = February 01 - February 15, 2020, a. s. o., Mar March, Apr April, PD Parkinson’s disease, HY Hoehn and Yahr stage, COVID-19 coronavirus disease 2019

| Diagnosis | ICD-Code | Case numbers | Jan B | Feb A | Feb B | Mar A | Mar B | Apr A | Apr B | May A | Sum Jan-May | Difference Mar A to B 2020 | Difference Apr A to B 2020 |  |
| --- | --- | --- | --- | --- | --- | --- | --- | --- | --- | --- | --- | --- | --- | --- |
| Any | All ICD | 2019 | 899,103 | 820,147 | 709,363 | 799,902 | 697,087 | 843,684 | 708,182 | 776,623 | 6,254,091 | -27.80% | 20.50% |  |
|  |  | 2020 | 881,798 | 775,588 | 742,015 | 743,158 | 536,243 | 440,347 | 530,459 | 560,824 | 5,210,432 |  |  |  |
|  |  | Diff. | -17,305 | -44,559 | 32,652 | -56,744 | -160,844 | -403,337 | -177,723 | -215,799 | -1,043,659 |  |  |  |
|  |  | Diff. % | -1.90% | -5.40% | 4.60% | -7.10% | -23.10% | -47.80% | -25.10% | -27.80% | -16.69% |  |  |  |
| COVID-19 | U07.1 | 2019 | 0 | 0 | 0 | 0 | 0 | 0 | 0 | 0 | 0 | 623.00% | -50.50% |  |
|  |  | 2020 | 28 | 62 | 190 | 1,628 | 11,770 | 10,148 | 5,026 | 1,975 | 30,827 |  |  |  |
|  |  | Diff. | 28 | 62 | 190 | 1,628 | 11,770 | 10,148 | 5,026 | 1,975 | 30,827 |  |  |  |
|  |  | Diff. % | 100% | 100% | 100% | 100% | 100% | 100% | 100% | 100% | 100% |  |  |  |
| PD | G20 | 2019 | 2,333 | 1,982 | 1,829 | 1,953 | 1,925 | 2,046 | 1,771 | 2,015 | 15,854 | -60.30% | 65.70% |  |
|  |  | 2020 | 2,232 | 1,950 | 1,887 | 1,932 | 767 | 559 | 926 | 1,009 | 11,262 |  |  | |
|  |  | Diff. | -101 | -32 | 58 | -21 | -1,158 | -1,487 | -845 | -1,006 | -4,592 |  |  | |
|  |  | Diff. % | -4.30% | -1.60% | 3.20% | -1.10% | -60.20% | -72.70% | -47.70% | -49.90% | -28.96% |  |  | |
| HY <3 | G20.0- | 2019 | 260 | 266 | 237 | 230 | 244 | 278 | 216 | 242 | 1,973 | -68.40% | 50.70% | |
|  |  | 2020 | 267 | 253 | 250 | 237 | 75 | 69 | 104 | 142 | 1,397 |  |  | |
|  |  | Diff. | 7 | -13 | 13 | 7 | -169 | -209 | -112 | -100 | -576 |  |  | |
|  |  | Diff. % | 2.70% | -4.90% | 5.50% | 3.00% | -69.30% | -75.20% | -51.90% | -41.30% | -29.19% |  |  | |
| HY 3-4 | G20.1- | 2019 | 1,603 | 1,322 | 1,208 | 1,322 | 1,266 | 1,339 | 1,216 | 1,354 | 10,630 | -58.70% | 63.00% | |
|  |  | 2020 | 1,542 | 1,320 | 1,296 | 1,305 | 539 | 346 | 564 | 615 | 7,527 |  |  | |
|  |  | Diff. | -61 | -2 | 88 | -17 | -727 | -993 | -652 | -739 | -3,103 |  |  | |
|  |  | Diff. % | -3.80% | -0.20% | 7.30% | -1.30% | -57.40% | -74.20% | -53.60% | -54.60% | -29.19% |  |  | |
| HY 5 | G20.2- | 2019 | 272 | 213 | 213 | 232 | 220 | 251 | 175 | 234 | 1,810 | -64.40% | 63.10% | |
|  |  | 2020 | 257 | 210 | 198 | 225 | 80 | 84 | 137 | 126 | 1,317 |  |  | |
|  |  | Diff. | -15 | -3 | -15 | -7 | -140 | -167 | -38 | -108 | -493 |  |  | |
|  |  | Diff. % | -5.50% | -1.40% | -7.00% | -3.00% | -63.60% | -66.50% | -21.70% | -46.20% | -27.24% |  |  | |
| Severity not specified | G20.9- | 2019 | 198 | 181 | 171 | 169 | 195 | 178 | 164 | 185 | 1,441 | -55.80% | 101.70% | |
|  |  | 2020 | 166 | 167 | 143 | 165 | 73 | 60 | 121 | 126 | 1,021 |  |  | |
|  |  | Diff. | -32 | -14 | -28 | -4 | -122 | -118 | -43 | -59 | -420 |  |  | |
|  |  | Diff. % | -16.20% | -7.70% | -16.40% | -2.40% | -62.60% | -66.30% | -26.20% | -31.90% | -29.15% |  |  | |

**Table S4:** COVID-19 prevalence in PD inpatients with different disease severity between January 16 and May 15, 2020

| PD ICD-Code | Disease severity | n (PD) | n (COVID-19) | COVID-19 prevalence |
| --- | --- | --- | --- | --- |
| G20 | Any | 64,434 | 693 | 1.1% |
| G20.0x | HY <3 | 9,564 | 80 | 0.8% |
| G20.1- | HY 3-4 | 20,565 | 202 | 1.0% |
| G20.2- | HY 5 | 3,495 | 50 | 1.4% |
| G20.9- | Not specified | 30,810 | 361 | 1.2% |
| all ICD | Not applicable | 5,210,432 | 30,872 | 0.6% |

**Table S5:** COVID-19 associated inpatient mortality in age groups of PD and non-PD inpatients between January 16 and May 15, 2020. PD Parkinson’s disease, COVID-19 coronavirus disease 2019

|  | COVID-19 | | | | | | | | | | |  | Mortality difference  PD vs. non-PD | |  | COVID-19 mortality risk  PD vs. non-PD | | |
| --- | --- | --- | --- | --- | --- | --- | --- | --- | --- | --- | --- | --- | --- | --- | --- | --- | --- | --- |
|  | Total | | |  | non-PD | | |  | PD | | |  |  |  |  |  |  |  |
| Outcome | Any | Died | Mortality |  | Any | Died | Mortality |  | Any | Died | Mortality |  | p value | χ²(1) |  | OR | 95% CI | |
|  |  |  |  |  |  |  |  |  |  |  |  |  |  |  |  |  | LB | UB |
| Total | 30,872 | 6,486 | 21.0% |  | 30,179 | 6,241 | 20.7% |  | 693 | 245 | 35.4% |  | <0.001 | 90.96 |  | 2.098 | 1.791 | 2.457 |
| <30 years | 1,266 | 8 | 0.6% |  | 1,266 | 8 | 0.6% |  | 0 | 0 | n.a. |  | n.a. | n.a. |  | n.a. | n.a. | n.a. |
| 30-39 years | 1,411 | 14 | 1.0% |  | 1,411 | 14 | 1.0% |  | 0 | 0 | n.a. |  | n.a. | n.a. |  | n.a. | n.a. | n.a. |
| 40-49 years | 2,220 | 57 | 2.6% |  | 2,219 | 57 | 2.6% |  | 1 | 0 | 0.0% |  | 0.871 | 0.03 |  | 0.000 | n.a. | n.a. |
| 50-54 years | 2,017 | 96 | 4.8% |  | 2,012 | 96 | 4.8% |  | 5 | 0 | 0.0% |  | 0.617 | 0.25 |  | 0.000 | n.a. | n.a. |
| 55-59 years | 2,564 | 192 | 7.5% |  | 2,557 | 191 | 7.5% |  | 7 | 1 | 14.3% |  | 0.493 | 0.47 |  | 2.065 | 0.247 | 17.238 |
| 60-64 years | 2,593 | 296 | 11.4% |  | 2,573 | 295 | 11.5% |  | 20 | 1 | 5.0% |  | 0.364 | 0.82 |  | 0.406 | 0.054 | 3.047 |
| 65-74 years | 5,277 | 1,054 | 20.0% |  | 5,180 | 1,035 | 20.0% |  | 97 | 19 | 19.6% |  | 0.923 | 0.01 |  | 0.976 | 0.588 | 1.618 |
| 75-79 years | 3,962 | 1,118 | 28.2% |  | 3,811 | 1,062 | 27.9% |  | 151 | 56 | 37.1% |  | 0.012 | 6.38 |  | 1.526 | 1.089 | 2.139 |
| ≥ 80 years | 9,562 | 3,651 | 38.2% |  | 9,150 | 3,483 | 38.1% |  | 412 | 168 | 40.8% |  | 0.257 | 1.28 |  | 1.120 | 0.916 | 1.370 |

**Table S6:** Mortality in PD inpatients between January 16 and May 15, 2020 vs. 2019

|  | 2019 | | |  | 2020 | | |  | Mortality difference 2020 <> 2019 | | |
| --- | --- | --- | --- | --- | --- | --- | --- | --- | --- | --- | --- |
|  | n (all) | n (dead) | Mortality |  | n (all) | n (dead) | Mortality |  | Relative diff. | p | χ²(1) |
| Total | 82,563 | 4,045 | 4.9% |  | 64,434 | 3,679 | 5.7% |  | 16.5% | <0.001 | 36.844 |
| <30 years | 17 | 0 | 0.0% |  | 10 | 0 | 0.0% |  | n.a. | n.a. | n.a. |
| 30-39 years | 48 | 0 | 0.0% |  | 53 | 0 | 0.0% |  | n.a. | n.a. | n.a. |
| 40-49 years | 420 | 1 | 0.2% |  | 317 | 0 | 0.0% |  | -100.0% | 0.385 | 0.754 |
| 50-54 years | 886 | 4 | 0.5% |  | 681 | 2 | 0.3% |  | -34.9% | 0.542 | 0.372 |
| 55-59 years | 1,716 | 16 | 0.9% |  | 1,417 | 16 | 1.1% |  | 21.1% | 0.429 | 0.625 |
| 60-64 years | 3,123 | 61 | 2.0% |  | 2,522 | 37 | 1.5% |  | -24.9% | 0.083 | 3.009 |
| 65-74 years | 15,568 | 415 | 2.7% |  | 12,029 | 391 | 3.3% |  | 21.9% | <0.001 | 16.720 |
| 75-79 years | 20,316 | 883 | 4.3% |  | 14,758 | 770 | 5.2% |  | 20.0% | <0.001 | 28.719 |
| ≥ 80 years | 40,468 | 2,665 | 6.6% |  | 32,647 | 2,463 | 7.5% |  | 14.6% | <0.001 | 53.477 |
